# Projecting range-wide sun bear population trends using tree cover and camera-trap bycatch data

**DOI:** 10.1371/journal.pone.0185336

**Published:** 2017-09-29

**Authors:** Lorraine Scotson, Gabriella Fredriksson, Dusit Ngoprasert, Wai-Ming Wong, John Fieberg

**Affiliations:** 1 Department of Fisheries, Wildlife and Conservation Biology, University of Minnesota, St Paul, Minnesota, United States of America; 2 Pro Natura Foundation, Balikpapan, East Kalimantan, Indonesia; 3 Conservation Ecology Program, King Mongkut’s University of Technology Thonburi, Bangkok, Thailand; 4 Panthera, New York, New York, United States of America; University of South Carolina, UNITED STATES

## Abstract

Monitoring population trends of threatened species requires standardized techniques that can be applied over broad areas and repeated through time. Sun bears *Helarctos malayanus* are a forest dependent tropical bear found throughout most of Southeast Asia. Previous estimates of global population trends have relied on expert opinion and cannot be systematically replicated. We combined data from 1,463 camera traps within 31 field sites across sun bear range to model the relationship between photo catch rates of sun bears and tree cover. Sun bears were detected in all levels of tree cover above 20%, and the probability of presence was positively associated with the amount of tree cover within a 6-km^2^ buffer of the camera traps. We used the relationship between catch rates and tree cover across space to infer temporal trends in sun bear abundance in response to tree cover loss at country and global-scales. Our model-based projections based on this “space for time” substitution suggested that sun bear population declines associated with tree cover loss between 2000–2014 in mainland southeast Asia were ~9%, with declines highest in Cambodia and lowest in Myanmar. During the same period, sun bear populations in insular southeast Asia (Malaysia, Indonesia and Brunei) were projected to have declined at a much higher rate (22%). Cast forward over 30-years, from the year 2000, by assuming a constant rate of change in tree cover, we projected population declines in the insular region that surpassed 50%, meeting the IUCN criteria for endangered if sun bears were listed on the population level. Although this approach requires several assumptions, most notably that trends in abundance across space can be used to infer temporal trends, population projections using remotely sensed tree cover data may serve as a useful alternative (or supplement) to expert opinion. The advantages of this approach is that it is objective, data-driven, repeatable, and it requires that all assumptions be clearly stated.

## Introduction

Management and conservation of species and sub-populations threatened with extinction requires accurate and reproducible estimates of population trend. Measuring changes in the global status of a species usually requires data collected over broad spatial and temporal scales. Yet when management resources are limited, monitoring programs tend to be restricted in scope, with data collected within a single study area over short periods, and with limited ability to extrapolate to other areas. Of the > 5000 mammalian species categorized by the International Union for the Conservation of Nature (IUCN) Red List of Threatened Species, > 800 are classed as data deficient (status unknown), and 25% are threatened with extinction. Red List assessments are often based on a combination of anecdotal data and expert opinion [1–3]. For some species, international collaborators combine data from multiple study sites to monitor populations on regional and global scales [4–7]. Population change is sometimes measured indirectly, using a proxy measure, such as change in habitat extent [8–10].

Sun bears *Helarctos malayanus* inhabit the tropical forests of Southeast Asia, use a broad spectrum of forest types, and select habitat based on food availability and security, favoring interior forest but also using secondary, logged and regenerating burnt forests [11–16]. Sun bears also feed in, and travel through non-natural vegetation, using agricultural areas close to the forest edge [16–19]. Populations are threatened by rapid deforestation [20,21], with steeper declines detected in areas of high deforestation compared with areas of low deforestation [22]. Global range is contracting, and local extirpations are possible within the northern range limits of Bangladesh and China, and extirpation may be imminent in Vietnam [20,23–25]. The IUCN classifies sun bears as vulnerable, estimating that populations have declined by ~35% in the past 30 years [20]. Data on global population trends for sun bears are deficient and studies that quantify the status of populations are few and limited to small areas. Density estimates are available for two national parks in Thailand [26] and population trends have been measured in one National Park in Sumatra [22] and several sites in Thailand [27,28]. Results of these studies cannot be readily extrapolated to other regions because they use model predictors that cannot be derived for areas outside those sampled, nor are they easily compared between sites because of differences in field methods. Faced with a lack of field data, the IUCN sun bear Red List assessment used opinions of a small number of expert representatives to determine risk [20].

The use of expert opinion and antidotal data to forecast population trends and to estimate risk of extinction generates a semi-subjective measure that is hard, or impossible, to replicate. Like many threatened species, sun bears would benefit from a more objective method for ongoing monitoring through time. If possible, monitoring methods should be feasible, inexpensive, and estimates of trend must be comparable between time periods (i.e. methods should stay consistent through time). As an alternative to expert opinion, changes in forest cover and rates of deforestation can be used to calculate changes in areas of occupancy and to infer population decline, by assuming that the rate of population change is equal to the rate of forest loss [8,9,29]. The IUCN Red List guidelines permits this measure as a viable alternative to estimating population trend in the absence of suitable field data, despite that habitat loss is not the only driver of abundance, and the relationship between habitat loss and population change is often not linear [30]. The recent availability of online, satellite-based tree cover change data collected between 2000 and 2014 [31], enables researches to tailor their measurements of deforestation rates to their specific systems (i.e. habitat change for forest dependent species). For example, previous studies have used spatial-temporal trends in forest loss as a surrogate for population declines by measuring discrete changes in tree cover within a species’ geographical and elevational range limits [8–10].

Here we explore whether data capturing tree cover loss may be used to quantify changes in sun bear populations through time. We develop a simple, replicable univariate model, relating sun bear presence to tree cover (i.e. habitat). Although many factors contribute to population trends, tree cover data are available range-wide and might be related to food availability, and perhaps also other underlying processes (shelter, security, human disturbance) that relate to mortality risk (factors for which data are not yet available in a uniform measure across the entire sun bear range). When long-term datasets are unavailable, as is the case for sun bears, patterns across space are sometimes used as a surrogate for patterns through time [32–34]. We used bycatch data pooled from multiple camera trap studies within sun bear range, and integrated models using detection/non-detection data and independent catch rate data. We used the relationship between tree cover and relative density of sun bears across space to project population change due to habitat loss. Using this “space for time” substitution, by assuming that the drivers of the spatial gradient between sun bears and % tree cover also drive temporal changes [35], we provide a standardized proximate measure of sun bear population change through time based on deforestation data, at least until better data become available. Data driven models have the benefit over expert opinion in that they can be repeated, and, as these models have explicit assumptions, they can also be debated, modified, and improved.

## Methods

To model the relationship between % tree cover and sun bear detections, we combined camera trap catch rates of sun bears, pooled from multiple study sites, with high resolution (30 x 30 m) tree cover measured on a continuous scale (0–100%). We used the spatial relationship between % tree cover and sun bear density to estimate temporal population declines associated with habitat loss between 2000 and 2014 within global sun bear range. To compare our estimates with the IUCN’s Red List classification for sun bears, we cast our estimates over a 30-year period, assuming a constant rate of tree cover change over that period.

### Sun bear detections at camera traps

**Fig 1:**
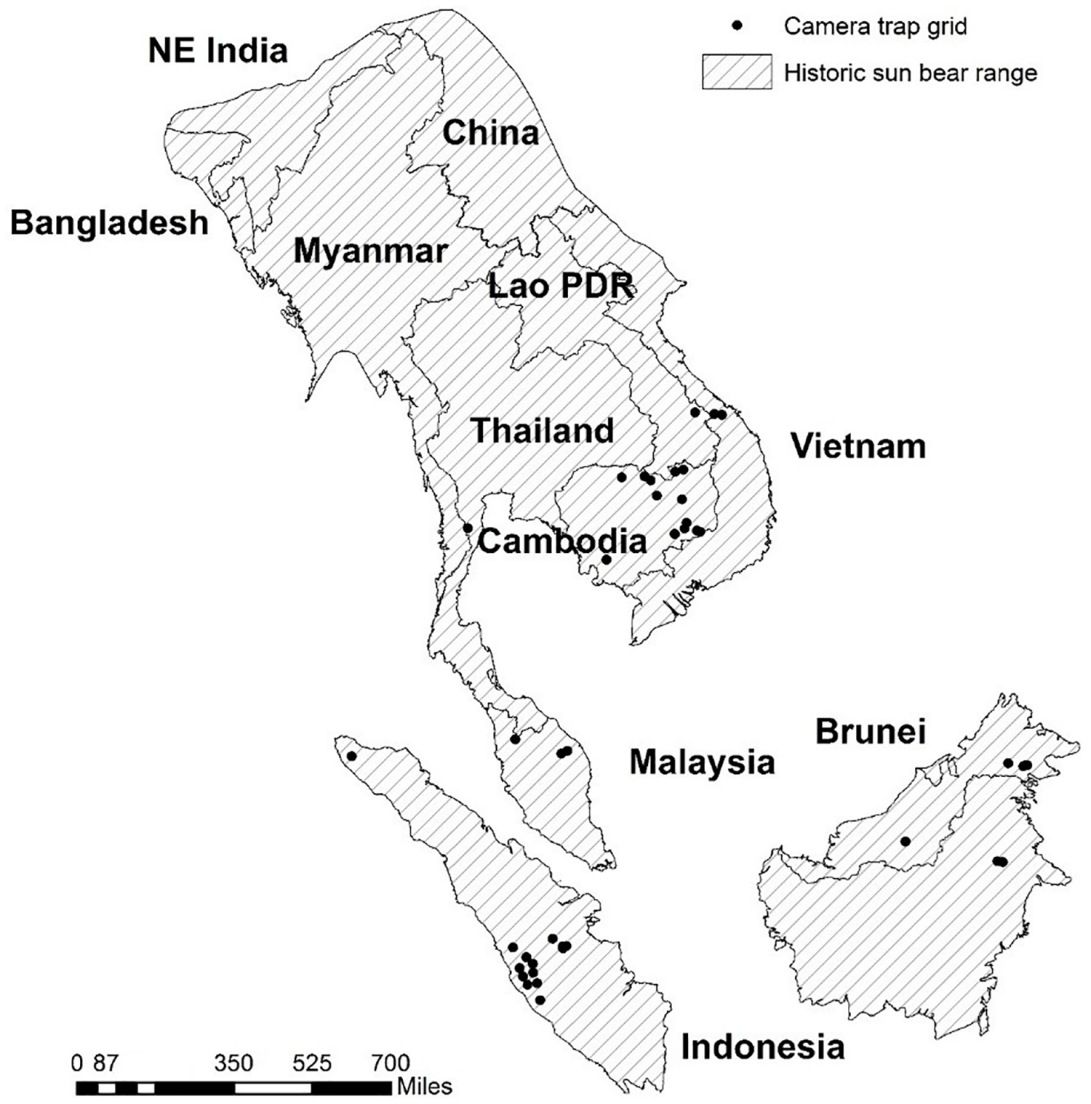
Sun bear range limits and distribution of camera trap field sites from which sun bear detection data were collected between 2000–2015. Historic (within 500 years) sun bear range extends southwards, from southeast Bangladesh, northeast India and southern China, throughout most of mainland southeast Asia, and all of Malaysia and Indonesia [23]. Camera trap data, collected between 2000–2015, were combined from 7 out of 11 sun bear range countries to project range-wide population trends using changes in tree cover between 2000–2014.

We obtained sun bear detection data from 49 non-baited camera-trap studies that were conducted within sun bear range between 2000 and 2014 (Fig 1; L.S., S1 Table). The primary objectives of these studies included biodiversity monitoring, and single species surveys (e.g. tiger *Panthera tigris* occupancy, Bornean orang-utan *Pongo pygmaeus morio* terrestrial behavior, sun bear occupancy), but cameras also captured sun bears and many other species. Camera trap metadata included a GPS location, the date the camera was set, and number of nights the camera was active (trap nights). For six of the 49 sites, only an average number of trap nights (across all cameras at the site) was available. We recorded the number of independent sun bear detections per camera trap (independent count data) at sites where camera data included timestamps for each picture, or, if time intervals between images were not known, the detection/non-detection of sun bears within a trapping period. To determine independent counts (when timestamps were available), we sorted photos using a minimum criterion for photo independence of one hour; sun bears are wide ranging, with an average daily movement of 2.7 km [11,13,16], and it was considered unlikely that a bear would stay around a camera trap for an hour or more (traps were not baited). Using this criteria of one hour removed all instances of multiple consecutive photos from of a bear hanging around a camera trap, and in the resulting database no detections occurred < 3 hours apart (most occurred > 1 day apart). For all camera trap data from the mainland region (study sites = 17, camera traps = 843), the number of trap nights was known but the time between sun bear detections was unknown and independent detections could not be determined. For these data, we recorded detection/non-detection within a known trapping period. If cameras were set in pairs, we systematically used the data from the second unit (i.e. in the order units were listed on the datasheet sent to us by data contributors). To reduce variability in sampling intensity among study sites, we filtered the data by removing camera units operational for < 7 days and > 3 months, and removed field sites with < 10 camera traps.

We tested for differences in sun bear response to % tree cover between the two major ecological regions of southeast Asia; the mainland (all countries north of Peninsular Malaysia) where climate is seasonal, and the insular region (Peninsular Malaysia, Sumatra and Borneo) where climate is aseasonal. The mainland data were collected in Thailand, Lao People’s Democratic Republic (here after Lao PDR) and Cambodia, and these data were assumed to be representative of all other countries in that region (Fig 1). China and Bangladesh were excluded from the analysis because sun bears may be extirpated in these countries, and the extent of historical range is unknown [20]. We analyzed data globally, with mainland and insular data combined, and regionally, with mainland and insular data analyzed separately (Table 1).

**Table 1:** Camera trap sun bear detection data, collected between 2000–2015, were combined from 31 field sites in 7 out of 11 sun bear range countries.

Region	Study sites	Camera traps (min/max per site)	Trap-nights (min/max per site)	Count^a^	Detections/total units^b^
Mainland	14	623 (13–126)	26,140 (8–90)	NA	379/623
Insular	17	843 (13–201)	48,752 (8–90)	524	246/843
Total	31	1,463 (13–201)	74,891 (8–90)	NA	379/2,022

^a^Independent sun bear detections per unit (independent count data); detections were considered independent if they occurred > 1 hour apart.

^b^For all camera trap data from the mainland region, the time-lag between sun bear detections was unknown, so we recorded detection/non-detection within a trapping period.

### Remote sensing data

To calculate tree cover at camera traps around the time they were active (between 2000 and 2015), we downloaded the three satellite-based tree cover rasters that were available at the time of writing from open source Global Forest Watch (www.globalforestwatch.org, accessed 14^th^ Feb 2017). Tree cover is any vegetation above 5-m height and does not distinguish between habitat types or natural and non-natural vegetation. Tree cover (%) reflects differences in habitat assemblages, and is highest in tropical evergreen forests, lower in secondary degraded forest, and lowest in lowland dry dipterocarp forest (S3 Fig). Tree cover within non-natural vegetation (e.g. agriculture, rubber and palm oil plantations) usually falls below 20% (S4 Fig). The three rasters we downloaded were; i) tree cover for the year 2000 (pixels valued from 0–100% tree cover), ii) tree cover loss from between 2000–2012 and iii) tree cover loss from between 2000–2014 (pixels of loss rasters were valued 1 [100% loss of tree cover within pixel] or 0 [no loss]. We trimmed all rasters to the geographic extent of historic sun bear range [23]. We created tree cover rasters for 2012 and 2014 by masking out tree cover that was lost by the years 2012 and 2014 by i) multiplying all loss pixels by 100 to transform pixel values to be 0 or 100, and on the same scale as the tree cover layer and ii) subtracting the transformed loss raster from the year 2000 tree cover raster. All negative values, when 100% loss was subtracted from a cell with < 100% tree cover, were transformed to zero. We did not incorporate tree cover gain, which is also available from Global Forest Watch, because this includes an unknown amount of planted forest, which tends to be intensively managed single species plantations (e.g. eucalyptus, teak, rubber; [36]) and not viable for sustaining bear populations. We smoothed the tree cover rasters for all years by averaging pixel values over a 6-km^2^ circular area (circular radius = 1.38 km), wide enough to represent the area of a core sun bear home range (home range estimates of sun bears range from 4–27.5 km^2^; [11,13,16] and narrow enough to maintain variability in tree cover within the scale of a camera trapping site. We processed Global Information System (GIS) data in ArcGIS 10.2.

## Data analysis

### Relating camera trap catch rates to tree cover

To investigate if sun bear detection rate was associated with percent tree cover, we extracted the average percent tree cover within a 6-km^2^ circular area at each camera trap location. Tree cover values were drawn from whichever raster (2000, 2012, or 2014) was closest in time to when the camera was active. The 2000 raster was used for units active before 2006, 2012 for units active from 2007–2012, and 2014 for units that were active post 2012 (tree cover changed very little at camera trap sites over the course of the study period; S1 Fig).

**Fig 2:**
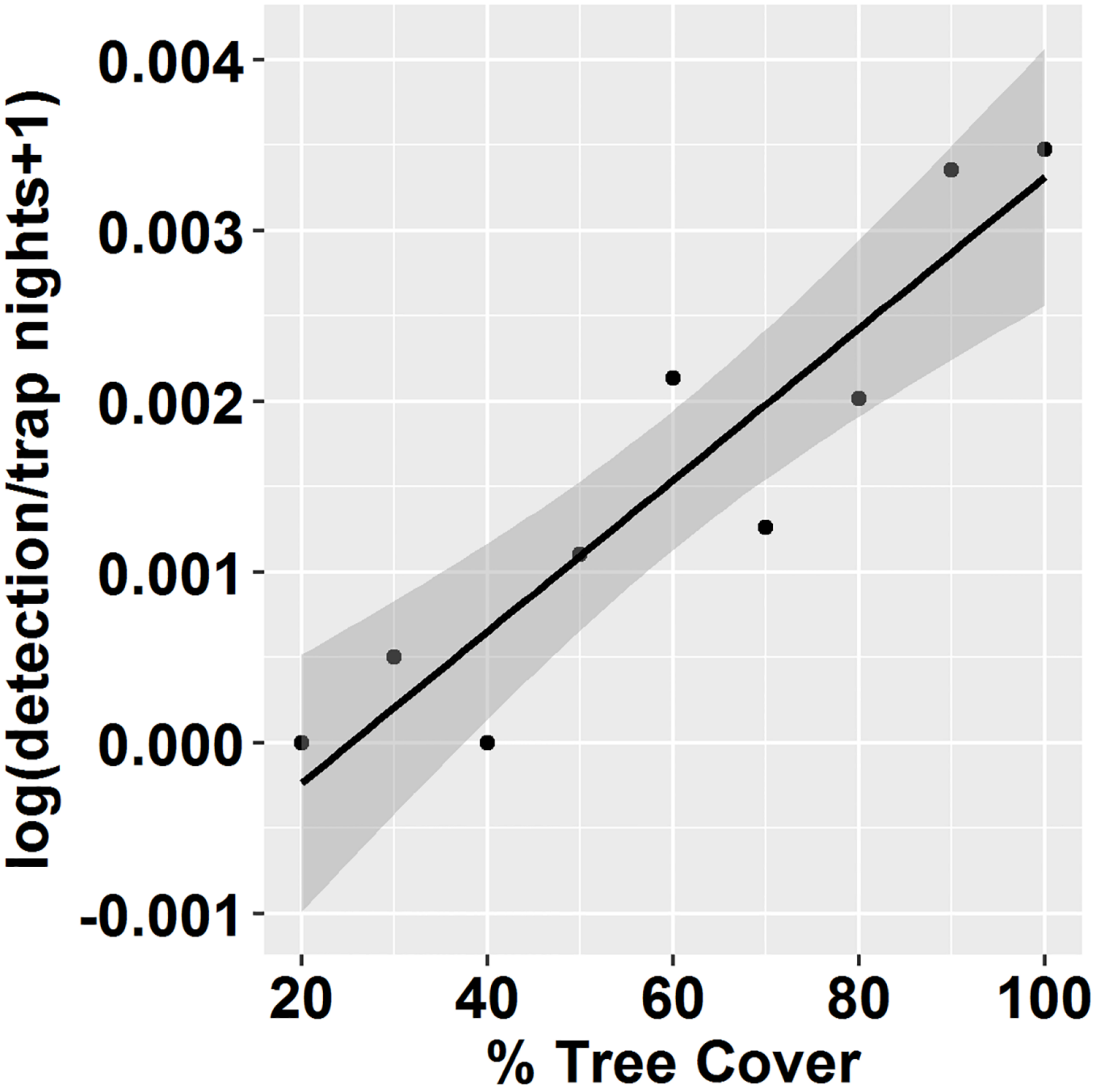
The log-linear relationship between sun bear detection rates at camera traps and % tree cover. We pooled data from camera traps active between 2000–2015 into % tree cover categories (<20%, 21–30, 31–40, …91–100), and calculated the detection rate of sun bears within each category by dividing the total number cameras that detected a sun bear at least once within a trapping period by the total number of traps nights cameras were active. We log-transformed detection rates and increased by 1 to avoid infinite values. Camera traps were active within all levels of tree cover, and were more active in areas of high tree cover (S2 Fig). Tree cover (0–100%) at camera traps, taken from rasters of tree cover closest in time to when cameras were active, were averaged over a 6-km^2^ area around camera traps to represent tree cover at the scale of a core sun bear range. In a simple linear regression, sun bear detections (log) rates were positively related with % tree cover (ln([Y]/Trap Nights+1) = -8.16 + 0.03*Tree Cover, R^2^ = 0.7).

To visually explore the data prior to model fitting, we pooled data from camera traps into % tree cover categories (<20%, 21–30, 31–40, …91–100), and calculated the detection rate within each category by dividing the total number of cameras that detected a sun bear at least once during a trapping period by the total number of traps nights cameras were active. We plotted the log(detection rate + 1) versus % tree cover and overlaid the fit of a simple log-linear regression relating these two variables (Fig 2).

We analyzed data separately by region to allow for potential differences in the response of bears to tree cover in the mainland and insular regions. For camera traps in the insular region, we modelled the relationship between the expected catch rate (number of independent camera detections/number of trap nights) as a log-linear function of % tree cover:

log(E[Yi]/trapnightsi)=β0+β1%TreeCoveri, (1)

where E[*Y*_*i*_] represents the expected number of detections at site *i* [37]. For the mainland, for which independent catch rate data were not available, we fit binary regression models to the detection/non-detection data (Z_i_ = 1 if detected at site *i* and 0 otherwise), using a complimentary log-log link:

log(−log(1−E[Zi]/trapnightsi))=β0+β1%TreeCoveri, (2)

For count data that are Poisson distributed, the probability of a count ≥ 1 = 1- exp(λ), where λ is the mean of the Poisson distribution. Thus, a complementary log-log link provides a way to model Poisson distributed count data that have been reduced to presence-absence data–i.e, the slope coefficients in the count data and presence-absence data models should be equivalent in this case (see e.g., [38]). We fit both models using the glm function in Program R [39], with family = poisson(link = “log”) when analyzing the count data and family = binomial(link = “cloglog”) when modeling the detection/non-detection data. In Eqs 1 and 2, variable-length trapping periods across the different sites is accommodated by including an offset equal to log(trap nights) [37].

We used a cluster-level bootstrap, (resampling clusters [study sites] with replacement; sites had a minimum of 10 camera traps) to estimate uncertainty in the population-level relationship between tree cover and catch rate or detection/non-detection rate. This allowed us to relax the assumption that the counts were independent and Poisson distributed. Data collected from a single study site (or adjacent sites within a contiguous block of forest), by a single research team, were treated as a cluster of correlated observations. Observations from different field sites were treated as independent clusters. We refit models to 50,000 bootstrapped data sets. We treated the data regionally (mainland and insular separately) and globally (mainland and insular combined) to test for differences in response to tree cover between regions, and to capture uncertainty in our estimates of *β*_1_. In the latter case, we reduced the insular data to presence-absence records and fit the binary regression model given by Eq 2 to the combined (insular and mainland) data. We interpreted *β*_1_’s in terms of the "relative risk” of detecting bears at camera traps within 80% tree cover versus 20% tree cover [40]:

$$risk\;ratio\;=\frac{\text{exp}\left(0.8*\hat{\beta }1\right)}{exp\left(0.20*\;\hat{\beta 1}\right)}.$$ (3)

### Estimating sun bear population decline between 2000–2014

We used changes in tree cover within sun bear range to project potential population declines, assuming the relationship between detection rate and forest cover across space can be used to infer trends in bear density over time following tree cover loss. As with most species distribution models, we assume regression parameters capture spatial variability in the relative density of individuals (e.g., [41]). This assumption requires that the probability of detecting bears, when present, is similar across the range of tree cover values (if detection is lower in areas of dense forest, estimators of regression coefficients would be biased low). We further assume the population distribution is in equilibrium (i.e., that the number of bears is relatively constant within the *surveyed* habitat at the time it was surveyed [trapping periods were limited to < 3 months]) and that future changes in forest tree cover will result in similar relative and absolute densities of bears for any given level of forested tree cover.

Given these assumptions, we can estimate relative changes in absolute abundance (*N*) using:

ΔN=exp(β1[TreeCover2014−TreeCover2000])/exp(β1TreeCover2000), (4)

which does not depend on any unknown parameters.

We used the estimated regression coefficient, ${\hat{\beta }}_{1}$ from the global model (mainland and insular data combined), and Eq 4, to project the % change in the number of sun bears, regionally and by country (excluding China and Bangladesh), between 2000 and 2014 based on changes in % tree cover during that period. We used the bootstrap distribution of regression coefficients to calculate percentile-based 95% confidence intervals for these trend estimates. To generate estimates comparable with the IUCN classification of sun bears, we projected sun bear population decline between 2000–2030, following the IUCN’s guidelines of assuming the annual rate of % tree cover change remained constant through time [30].

## Results

### Relationship between sun bear detections at camera traps and % tree cover

**Fig 3:**
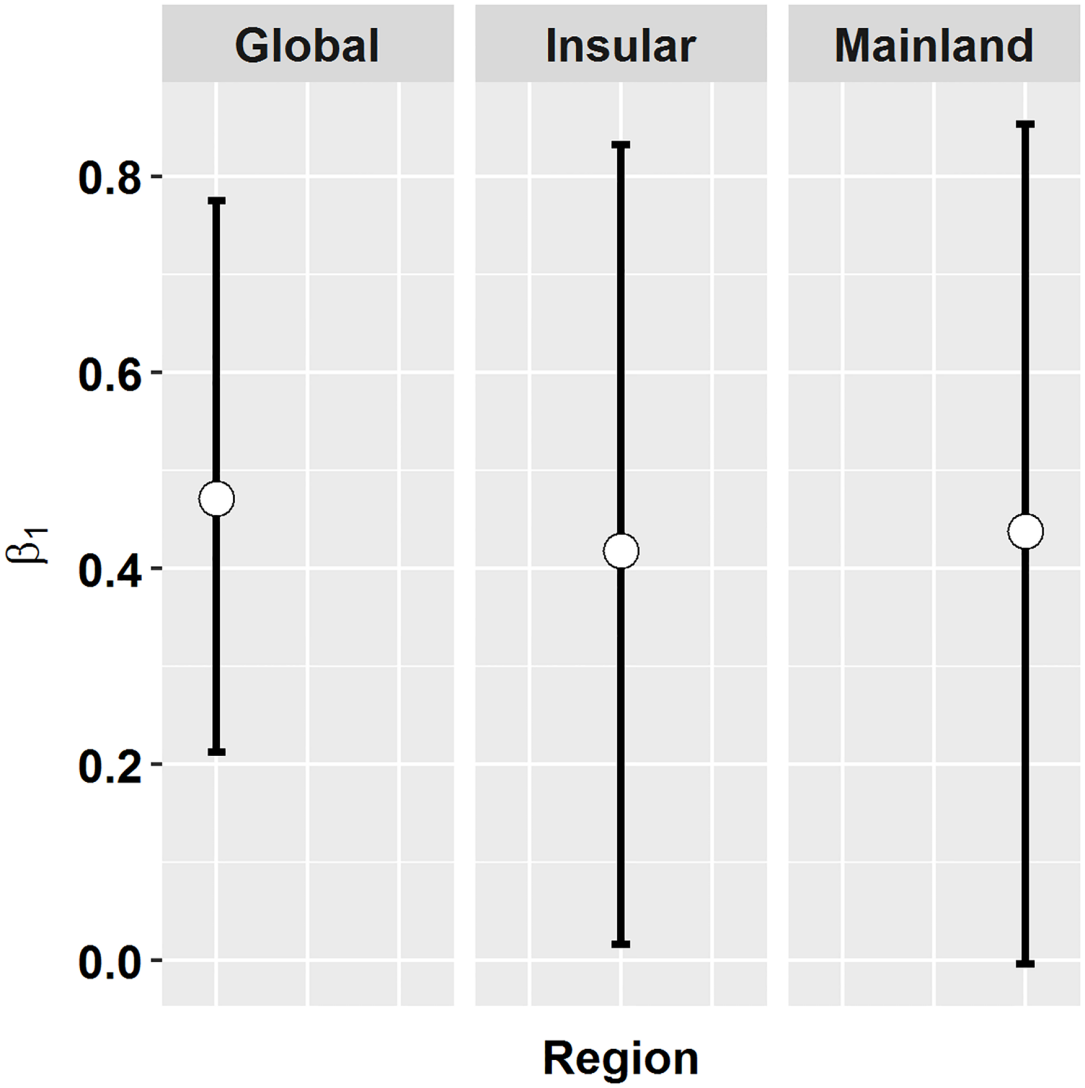
Estimated regression coefficients and 95% bootstrap confidence intervals for *β*_*1*_, relating the log expected catch rate to % tree cover. Regional models were fit to data from either the insular and mainland countries, allowing the response of sun bears to tree cover to vary by region. The insular data, catch rate per camera trap, were modelled using log-linear regression (Eq 1) and the mainland data, detection/non-detection per camera trap within a trapping period, were modelled using a binary regression model with complementary log-log link (Eq 2). The global model pooled all data, assuming bears responded similarly to tree cover throughout the range, and modelled detection/non-detection per camera trap within a trapping period using the binary regression model (Eq 2). Models assumed log catch rate of sun bears at camera traps was a linear function of tree cover averaged over a 6-km^2^ circular area surrounding the camera traps. Data were filtered to reduce variability in sampling intensity, by removing cameras active for < 7 days and > 90 days, and study sites with < 10 cameras.

Sun bears were detected in all levels of tree cover above 20% and detections were higher in areas of high % tree cover (Fig 2). In a simple linear regression, log-detection rates at camera traps were positively correlated with binned % tree cover data (R^2^ = 0.7; Fig 2). For the mainland model ${\hat{\beta }}_{1}$ = 0.44, 95% CI = -0.003–0.85, and for the insular model ${\hat{\beta }}_{1}$ = 0.42, 95% CI = 0.02–0.83. Because the ${\hat{\beta }}_{1}$ estimates (and CI’s) were similar between regions, we combined data from the two regions and refit the binary regression model (Eq 2), giving ${\hat{\beta }}_{1}$. = 0.47 (95% CI = 0.21–0.78; Fig 3). Using the combined model, the risk ratio (Eq 3) of finding a bear in 80% cover versus 20% cover was 1.46 (95% CI = 1.18–1.82). We used the combined model to project population trends over time following tree cover loss.

### Projected population declines

**Fig 4:**
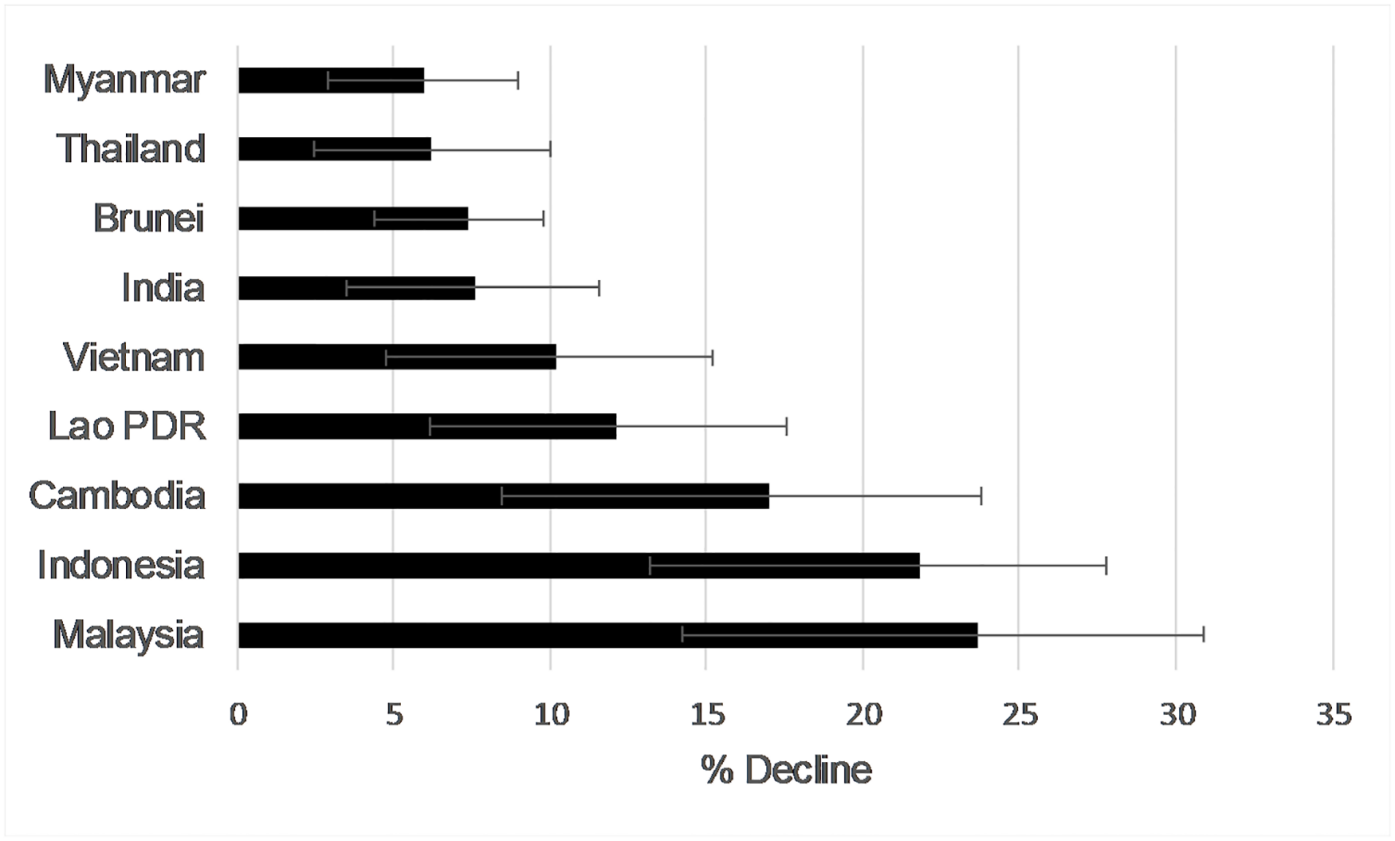
Model-based projections of sun bear population change across southeast Asia between 2000–2014. Bars, with 95% confidence intervals, show estimates generated by binary regression models (Eq 2) fit to the pooled mainland and insular data. Models assumed log catch rate of sun bears at camera traps was a linear function of % tree cover averaged over a 6 km^2^ circular area around camera traps. Country-level declines were predicted to be the most severe in Malaysia and Indonesia, which form the bulk of the insular region. On the mainland, declines roughly follow a longitudinal gradient, being highest in eastern countries (Cambodia, Lao PRD, Vietnam) and lowest in the west (India, Thailand, Myanmar).

**Fig 5:**
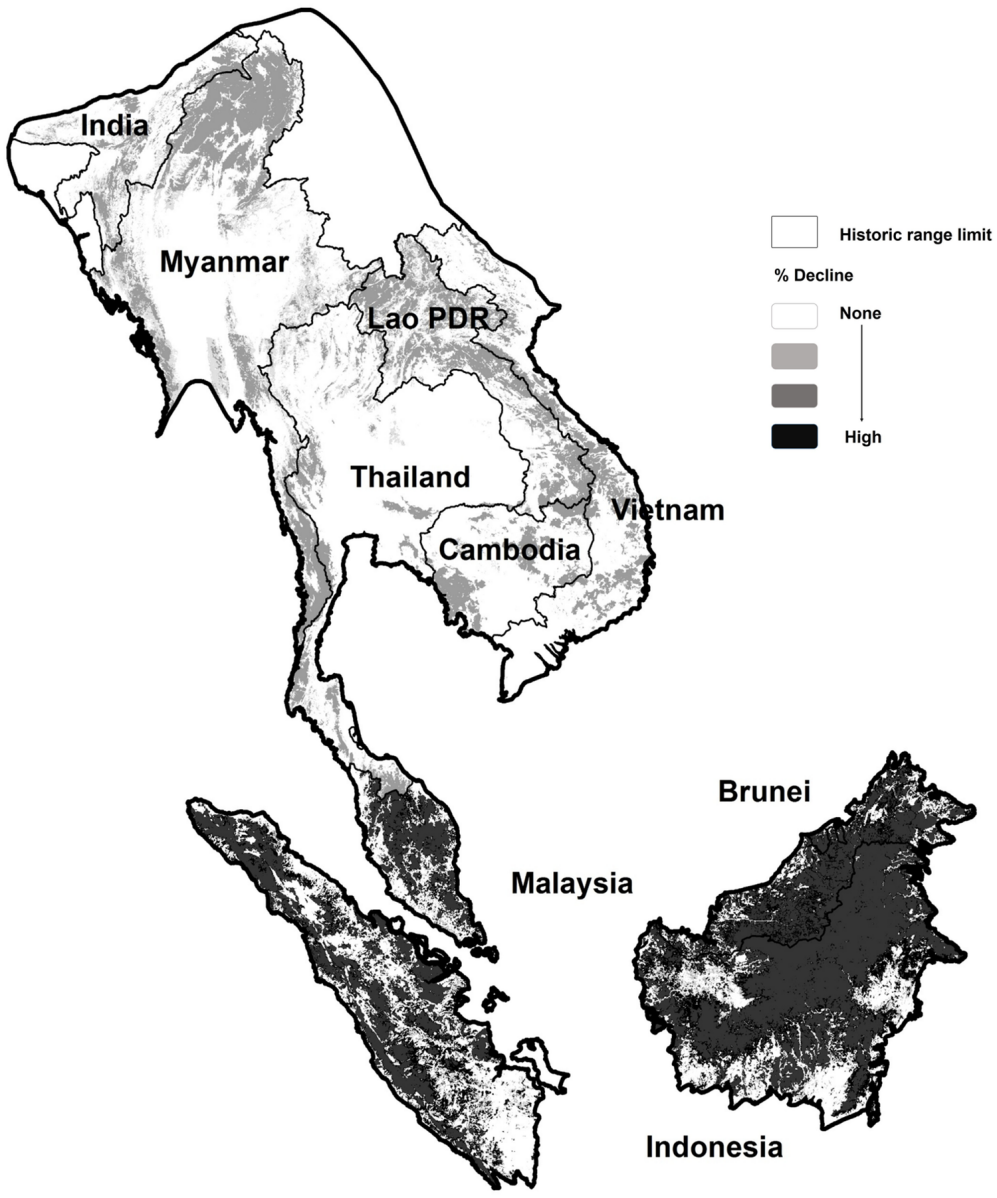
Projected country-level sun bear population declines between 2000–2014, based on the modelled relationship between sun bear catch rate at camera traps and % tree cover. Models assumed log catch rate of sun bears at camera traps was a linear function of % tree cover averaged over a 6-km^2^ circular area around camera traps. Country-level decline estimates are also summarized in Fig 4.

Population declines were projected to be highest in Indonesia and Malaysia, and lowest in Myanmar (Fig 4). The insular region was projected to experience a higher relative level of decline than the mainland region (Fig 5). Projected sun bear population losses over a 14-year period, between 2000–2014, were 22% (CI = 13.4–28.5) in the insular region, and 8.6% (CI = 4.1–12.9) on the mainland. Cast over 30 years, following the IUCN guidelines by assuming response to habitat loss is linear and that tree cover loss continues at a constant rate, projected sun bear decline in the insular region was almost 50% ($\bar{\text{x}}$ = 47.7, CI = 28.7–61.2) and approached 30% on the mainland ($\bar{\text{x}}$ = 18.4, CI = 8.7–27.7).

## Discussion

Sun bear catch rates at camera traps were positively correlated with % tree cover. This relationship was expected, and suggests that % tree cover is related to factors that drive sun bear abundance. Because sun bears are forest dependent with a broad spectrum of habitat use, the tree cover variable, which encompasses all habitat types, may be an appropriate proximate measure of population change until something better becomes available. Population declines associated with changes in habitat extent and quality could be driven by several cumulative and inter-related mechanisms that reduce reproductive rates and access to resources, and increase mortality [42–44]. Higher tree cover may be associated with more food resources, further distance to forest edges, and lower accessibility by humans (e.g. hunters, gatherers, researchers, loggers). As such, tree cover may encompass several predictors or underlying processes that influence bears, much like greenness, a satellite derived measure of vegetation reflectance that has strong predictive power in modelling grizzly bear population dynamics [45]. The use of habitat change to directly infer population change is currently one of the best available options for monitoring many threatened species for which long-term data are not available [30]. For forest dependent species, such as sun bears, changes in tree cover over time, derived from satellite imagery, provides a standardized, model-based, objective and reproducible method to monitor changes in global populations through time, and a data driven alternative to using expert opinion to estimate population trends.

In the absence of temporal data on the effect of tree cover change on sun bear populations, we substituted “space for time” [32,35], assuming that the relationship we found between sun bear presence and tree cover across space can serve as a proxy for changes that might occur over time following tree cover loss. At the country-level, our projected 14-year (2000–2014) declines were highest in the insular countries of Indonesia and Malaysia (including Peninsular Malaysia). Brunei was an outlier, with a low level of decline compared with the rest of the insular region. On the mainland, north of the Isthmus of Kra, our projected rates of decline followed a longitudinal gradient, being highest in the eastern countries (Cambodia, Lao PDR, Vietnam) and lowest in the west (India, Thailand, Myanmar, Fig 4). Grouped regionally and cast over 30-years, in order to compare our estimates with the IUCN Red List status assessment for sun bears, the 95% confidence intervals of our insular projections overlapped with the IUCN expert-derived global estimates of sun bear population decline of ~ 35% for the past 30 years, while the upper limit of our mainland estimate is slightly lower [20]. Notably, in the insular region, the upper confidence limits of our predictions meet the Red List criteria for endangered, based on a declining population trend of > 50% over a 30-year period. These projections should be treated with caution as they rely on a number of assumptions, which we discuss in the sections below. Our wide bootstrap confidence intervals also reflect the unmeasured variation in local conditions, and serve as a helpful reminder that our models should not be used for fine scale interpretations, but are expected to capture broad, landscape-scale patterns.

### Limitations

Although our model-based approach is objective and replicable, it has many limitations that should be addressed in future work. We rely on a single quantifiable variable (i.e. tree cover) to represent all influencers of sun bear abundance. Like other studies that use change in habitat extent to infer population change, we do not consider the biological adaptiveness of sun bears to changing tree cover. Furthermore, sun bear populations respond to more than just tree cover, and site-level variation in bear population status, and the associated pressures (e.g. hunting, resource availability, competition), is unaccounted for in our models. An important sampling limitation is that most camera trap sites were within protected areas (S1 Table) and did not undergo much change in tree cover during the time scale of our analysis (S1 Fig), therefore our sample may not be representative of the non-protected areas included in our temporal analysis. We expect that hunting by humans is a major determinant of local population status. Bears affected by mortality pressure in habitats below carrying capacity might not be affected by forest loss because they are already reduced below food potential (e.g. ‘empty forest syndrome; [46]). Given the high value of sun bear gall bladders, paws, other body parts and cubs, and widespread deficiencies in law enforcement capacity within sun bear range, we expect that levels of hunting are generally high in most areas [47,48]. Local population status may be also affected by differences in resource patterns and competitive pressures between the mainland and insular regions, caused by varying climate (seasonal versus a-seasonal forest) and species assemblages (e.g. presence/absence of the larger Asiatic black bear). Yet, the regression parameters describing the effect of tree cover on catch rate were similar for the mainland and insular models, leading us to pool data. Pooling the data led to narrower confidence intervals and a slightly higher estimate of *β*_*1*_ (the small change in the estimate is attributable to the pooling of data and reduction of the insular data to detection/non-detection data so that a common binary regression model (Eq 2) could be applied in the global model). Our model-based approach has the main benefit of being replicable, and with explicit assumptions that can be stated, debated, modified and potentially improved in future studies. This relationship, and our results, are valid only to the extent that several important assumptions hold true.

In the absence of long-term data sets that measure sun bear’s response to changes in % tree cover through time, a key assumption in our approach is that it was reasonable to substitute spatial relationships for temporal ones [32,35]. We found that the relative risk ratio of finding bears in 80% tree cover versus 20% tree cover was 1.46. Applied to a temporal trend, this suggests that if a patch of 80% tree cover was degraded into 20% tree cover, bear density would be reduced by 46%. This space-for-time assumption works well in some cases, such as in measuring the response of bluefin killfish to environmental change [33] and predicting the effects of climate-change on biodiversity [35], but has underestimated the consequences of environmental change in others [34], and may perform poorest when there is high spatial variation on conditions that affect the response [35]. Nevertheless, using spatial gradients to infer temporal changes is particularly useful for managers with limited resources and no history of maintaining long-term monitoring data on species of interest [33]. The space-for-time assumption is common in published assessments of biodiversity and ecosystem functioning, but is not always reliable; to test whether our assumption of space-for-time holds, and that our model projections are reasonable, a future study should ideally test the response of sun bear populations to tree cover with before-after control data (i.e. [34]).

Another key assumption is that sun bear detection rate is related to bear density. Many studies assume detection rates are related to density, [49,50], including camera trap studies [51], and this assumption underpins distance sampling methodology [52]. Nonetheless, we are aware there are caveats that influence this relationship. In particular, relative use of different habitats, and thus measures of habitat suitability or selection, will vary with both habitat availability and population density [53–55]. Population dynamics within agricultural forests (palm oil and rubber plantations) will undoubtedly vary from patterns within natural forests. Finally, our models assumed a positive relationship between sun bear population change and tree cover change, and that bears will not simply redistribute their numbers within the landscape when % tree cover changes.

Violation of these assumptions is inevitable to some degree, in some cases inflating our estimates, and in others causing underestimates of decline. Un-modelled effects from hunting and habitat fragmentation pressures may cause higher declines than we have estimated. Conversely (but perhaps too optimistically), failure to incorporate the adaptiveness of sun bears to a changing landscape may cause inflated estimates. The declines predicted by our models are unlikely to be instantaneous, and bears presumably did redistribute themselves in the landscape in the short-term, with an unknown time-lag between when deforestation occurred and the resulting population declines. Our estimates are regional, and we did not combine them to create an over-all global measure of decline because sun bear densities may vary greatly between insular and mainland populations [20,56] and a simple combination would be biased and/or conceal a steeper decline in a significant part of sun bear range. Reliable density estimates of regional sun bear populations would allow an area/density weighted measure to generate more accurate decline estimates.

A full picture of sun bear status requires fine-scale knowledge on the status of sub-populations. Many desirable variables, including landscape, anthropogenic and biological measures were not available for inclusion. Future researchers might test, refine, and improve our analysis with the addition of a more informative suite of predictor variables, either at a small geographical scale, or across sun bear range as those data become available. Additional variables might include human disturbance variables such as human density, road density, levels and types of land use [57], forest types and their relative abundance of sun bear foods, agricultural lands and their relationship to forest cover and sun bear foods, spatial variation in hunting, predation and competitive pressure. Useful landscape variables include habitat fragmentation metrics, which in combination with sun bear movement parameters, could be used to investigate the role of range connectivity and movement potential in determining the ability of populations to respond to losses in tree cover. Incorporating information about local densities of sympatric Asiatic black bears, which might suppress local sun bear populations in mainland Southeast Asia in areas of high food availability [26,56], could improve estimates by accounting for species competition. Finally, a future analysis would benefit from the ability to distinguish between vegetation types, so that planted vegetation types not used by bears (e.g. eucalyptus, teak, rubber) can be eliminated when projecting population changes.

### Conclusions

Sun bears and other threatened species require long-term, systematic and standardized monitoring of population trends across space and time. In Europe and North America, most knowledge of population demographics of bears comes from genetic and telemetry studies (e.g. [58–60]). Aside from the restrictive expense of these techniques for researchers working on low budgets in the tropics, these methods have been difficult to employ with sun bears. Researchers have had difficulty in collecting viable hair samples from sun bears due to their short pelt [26] and in collecting scats, which persist for a very short time in the rainforest, and are rarely encountered [61,62]. Telemetry studies of sun bears have been challenged by very low capture rates [11,13,16] and data have been insufficient for estimating population density and trends. Until now, polling field biologists (i.e., expert opinion) has been the only method used to generate estimates of regional and global population trends for sun bears [20]. Other efforts, using structured village workshops [27] and mark-recapture estimates by individually identifying sun bear chest markings with camera traps [63], are best suited to small spatial scales and while successful in generating robust site-level estimates of population parameters, results cannot be extrapolated to other sites, and the logistics and associated costs (time, money) of these methods make it unlikely they could be conducted over a large enough area to monitor regional or global bear populations. The IUCN Red List assessment of sun bears relied on a small number of people, meaning that each person had a significant influence on the global estimate, particularly when only one representative answered for a large extent of the range. The free availability of tree cover data, a uniform measure of % tree cover and temporal change over 14 years, between 2000 and 2014, has created new research opportunities in studies related to forest loss and fragmentation (e.g. [9,44,64,65]). Tree cover may act as a viable substitute for causal variables (food availability, hunting pressure) that are not available on broad scales. Given the limitations associated with employing other monitoring methods over large areas, or in keeping them standardized through time, monitoring sun bears with satellite based tree cover change may be the most realistic long-term solution because these types of models can be applied repeatedly over time, as updates to tree cover data become available.

We have presented an alternative approach to that of expert-based estimates for monitoring the population trends of threatened species. We collected the largest catalogue of sun bear detection data to date, and made an objective estimate of global population change. Our study demonstrates the potential of using camera trap data to monitor threatened species even when most were collected on studies for which bears were not a primary focus. The conservation community would benefit greatly if more efforts were made to systematically classify and manage camera trap imagery for use on a variety of topics [66]. Our approach could be repeated for other cryptic forest dependent species for which optimal habitat may be reflected in selection for tree cover, such as clouded leopard Neofelis spp. or Asian tapirs *Tapirus indicus*.

## Supporting information

S1 Table Camera trap field sites and identity of data contributors to a range wide analysis of sun bear population trends. (DOCX)

S1 Fig Changes in % tree cover between 2000–2014 at camera trap units. (PNG)

S2 Fig Rate of sun bear detections (detections/trap nights) at camera traps set between 2000–2015 within varying levels of % tree cover. (JPG)

S3 Fig Distributions of tree cover (%) found in primary tropical forest, dry dipterocarp forest and secondary forest (forest types collected from transect data in Lao PDR). (JPEG)

S4 Fig An example of tree cover (%) found in palm oil (Borneo) and agricultural (Peninsular Malaysia) plantations (tree cover values extracted to 1000 random points generated within each GIS layer). (PNG)

S1 Dataset Co-author contributed data (W.M.W). (CSV)
