# Supplementary material for: Projecting range-wide sun bear population trends using tree cover and camera-trap bycatch data
Source: PLoS One. 2017 Sep 29;12(9):e0185336. doi: 10.1371/journal.pone.0185336 (PMC5621681; doi:10.1371/journal.pone.0185336)
Supplement: S1 Table — (DOCX) [file pone.0185336.s001.docx]

S1. Camera trap field sites contributed from across Southeast Asian sun bear range.

| Study Area (Lat/Long)^1^ | Date | Area (Km^2^)^2^ | No. of Units | Contributor information |
| --- | --- | --- | --- | --- |
| Seima Protection Forest, Cambodia (107° E, 12 °N) | 2003 | 123 | 53 | Wildlife Conservation Society |
| Phnom Prich Wildlife Sanctuary, Cambodia (106.5° E, 12.5 °N) | 2001-07 | 2,113 | 119 | Thomas Gray, World Wide Fund for Nature |
| Siem Bok Forest, Cambodia (105.5° E, 13.5 °N) | 2001-06 | 826 | 32 | Thomas Gray, World Wide Fund for Nature |
| Siem Pang Forest, Cambodia (106° E, 14 °N) | 2003 | 5 | 7 | Thomas Gray, World Wide Fund for Nature |
| Virachey National Park, Cambodia (106.5° E, 14 °N) | 2001 | 566 | 9 | Thomas Gray, World Wide Fund for Nature |
| Seima Protection Forest, Cambodia (107° E, 12 °N) | 2004 | 125 | 7 | Thomas Gray, World Wide Fund for Nature |
| Tembat Forest Reserve,  Terengganu, Peninsular Malaysia (101 – 103° E, 5 – 5.5 °N) | 2011-12 | 437 | 452 | Reuben Clements |
| Kerinci Seblat Tropical Rainforest Heritage, Sumatra (8 sites; 101 – 102° E, 1.5 – 2.5 °S) | 2010-11 | 123 | 739 | Wai Ming Wong |
| Seima Protection Forest, Cambodia (107° E, 12 °N) | 2000-02 | 403 | 92 | Wildlife Conservation Society |
| Wehea Forest, East Kalimantan, Indonesia (116° E, 1.5 °N) | 2012-13 | 63 | 95 | Brent Loken |
| Batang Hari Forest, Sumatra (101° E, 1 °S) | 2008-09 | 196 | 21 | Yoan Dinata, Fauna & Flora International |
| Bukit Tiga Puluh National Park, Sumatra (102° E, 1 °S) | 2013-14 | 936 | 240 | [Alexander Moßbrucker](https://plus.google.com/u/0/115709515461971619671?prsrc=4), Frankfurt Zoological Society |
| Xe Sap National Protected Area, Lao PDR (107° E, 16 °N) | 2013 | 123 | 38 | Thomas Gray, World Wide Fund for Nature |
| Preah Vihear Protected Forest, Cambodia (105° E, 14 °N) | 2010-11 | 353 | 53 | Wildlife Conservation Society |
| Kuiburi National Park, Thailand (99.5° E, 12 °N) | 2007-12 | 291 | 88 | Rob Steinmetz |
| Kalabakan Forest Reserve, Sabah, Malaysia (4 sites; 117 – 117.5° E, 4.5 °N) | 2011-12 | 20 | 592 | Oliver Wearn |
| Ulu Masen Forest, Sumatra (96° E, 5 °N) | 2013 | 2,702 | 164 | Matt Linkie, Fauna & Flora International |
| Virachey National Park, Cambodia (106.5° E, 14 °N) | 2000 | 1,468 | 41 | Wildlife Conservation Society |
| Kirirom National Park, Cambodia (105.5° E, 14 °N) | 2000 | 933 | 46 | Wildlife Conservation Society |
| Kulen Promtep Wildlife Sanctuary, Cambodia (104.5° E, 14 °N) | 2010-11 | 419 | 37 | Wildlife Conservation Society |
| Hue and Quang Nam Saola Reserve, Vietnam (107.5° E, 16 °N) | 2012-14 | 210 | 127 | Thomas Gray, World Wide Fund for Nature |
| Cholong Forest, Phnom Veng, Cambodia (106° E, 12 °N) | 2003-05 | 32 | 17 | Thomas Gray, World Wide Fund for Nature |
| Khieu Forest, Prey Oso, Cambodia (106.5° E, 13 °N) | 2004-06 | 217 | 19 | Thomas Gray, World Wide Fund for Nature |
| Malaysian Borneo (11 sites; 114 – 115.5° E, 2 – 4 °N) | 2003-15 | 598 | 209 | Jayasilan Mohd-Azlan |
| Preah Vihear Protected Forest, Cambodia (105° E, 14 °N) | 2012-13 | 512 | 51 | Ai Suzuki |

*Footnotes:*

^1^Forest blocks are named primarily according the IUCN World Database of Protected Areas. Lat/Longs are approximate locations in decimal degrees.

^2^Minimum Convex Polygon drawn around perimeter of camera trap units.
