# Supplementary figures and images for: Projecting range-wide sun bear population trends using tree cover and camera-trap bycatch data
Source: PLoS One. 2017 Sep 29;12(9):e0185336. doi: 10.1371/journal.pone.0185336 (PMC5621681; doi:10.1371/journal.pone.0185336)

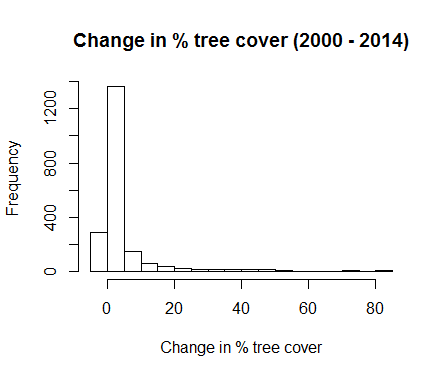

Supplement: S1 Fig — (PNG) [file pone.0185336.s002.png]

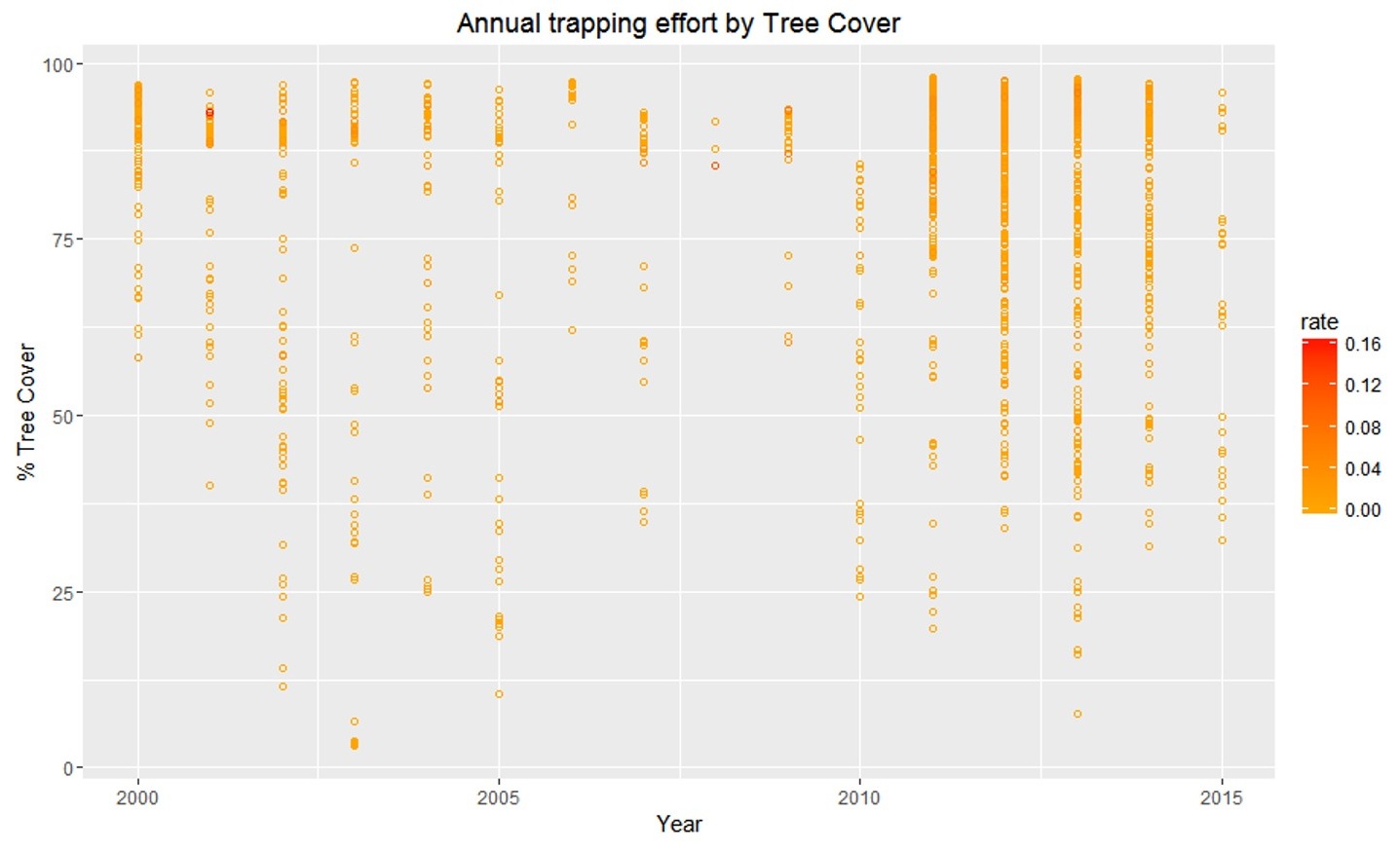

Supplement: S2 Fig — (JPG) [file pone.0185336.s003.jpg]

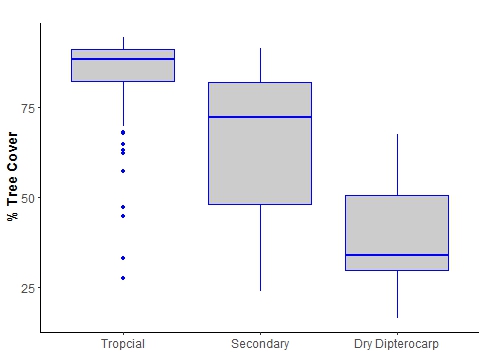

Supplement: S3 Fig — (JPEG) [file pone.0185336.s004.jpeg]

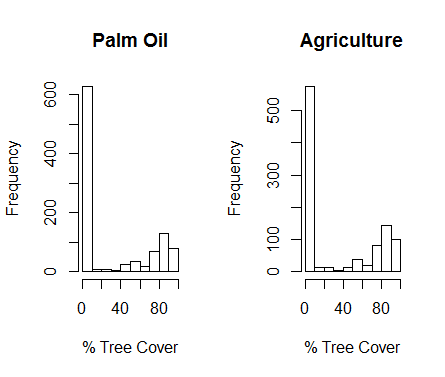

Supplement: S4 Fig — (PNG) [file pone.0185336.s005.png]
